# Development and application of an artificial intelligence-based quantitative system for white without pressure in myopic retinopathy

**DOI:** 10.3389/fcell.2026.1858522

**Published:** 2026-06-11

**Authors:** Pengcheng Wu, Lu Yang, Amei Wang, Yan Li, Yuqiu Zhang, Xin Zhao, Jie Wang, Yajie Zheng, Xingye Wang

**Affiliations:** 1 Department of Ophthalmology, The Second Hospital and Clinical Medical School, Lanzhou University, Lanzhou, China; 2 Gansu Province Clinical Research Center for Ophthalmology, Lanzhou, China; 3 Evision Technology (Beijing) Co., Ltd., Beijing, China

**Keywords:** artificial intelligence, high myopia, quantitative analysis, ultra-widefield imaging, white without pressure

## Abstract

White Without Pressure (WWOP) is a common but under-recognized peripheral retinal finding associated with myopia-related complications, yet its objective assessment remains challenging. This study aimed to develop and validate an artificial intelligence (AI) system for the automated segmentation and quantitative assessment of WWOP lesions from ultra-widefield color fundus photographs. The system employs deep learning algorithms for automatic identification and multi-dimensional quantification of WWOP lesions, including WWOP total area, total number, zonal distribution (Area and Number for R1 (posterior pole), R2 (mid-periphery), and R3 (far-periphery)), and global area ratio. Applied to 119 myopic eyes (64 with WWOP, 53.8%), the system revealed that over 96% of the lesion area resides outside the posterior pole, predominantly in the temporal and peripheral retina. Quantitative parameters showed significant positive correlations with axial length (e.g., total area: R = 0.41, P < 0.001; peripheral area R3: R = 0.40, P < 0.001; total number: R = 0.43, P < 0.001). This AI-powered system provides an objective, standardized, and high-throughput tool for WWOP evaluation but should currently be considered a research tool requiring further validation. Nevertheless, it holds strong potential to enhance clinical screening and risk stratification in myopia management.

## 1 Introduction

White Without Pressure (WWOP) is a common degenerative change in the peripheral retina of highly myopic eyes. It appears as non-depressed white or gray-white areas with irregular morphology, often progressing in an annular pattern near the vitreous base and ora serrata, particularly in the inferior retina (Syal et al., 2025; Karlin and Curtin, 1976; Fawzi et al., 2014; Khatwani et al., 2022). Given the global myopia epidemic, sight-threatening complications such as posterior staphyloma, choroidal neovascularization, and retinal detachment have emerged as major public health concerns (Du et al., 2024; Ikuno, 2017). Consequently, early identification and precise assessment of myopic retinopathy are crucial for delaying disease progression and preserving visual function. Epidemiological studies indicate that WWOP is highly prevalent in myopic populations, with its incidence positively correlating with the severity of myopia. In high myopia (spherical equivalent ≤ −6.00 D), the detection rate ranges from 25.4% to 51.7%, far exceeding the 5.8% rate in emmetropic controls, making WWOP one of the most common peripheral retinal findings in myopia (Zhang et al., 2018; Chen et al., 2018; Cheng et al., 2013; Elnahry et al., 2019). Specifically, in a cohort of office workers, WWOP was found in 19.7% of myopic eyes, with prevalence escalating from 10.9% in mild myopia to 43.8% in high myopia (Zhang et al., 2018). Notably, some patients may be unaware of the potential vision-threatening complications associated with myopia, such as those linked to WWOP, highlighting the need for enhanced screening and education (Zhang et al., 2018).

Traditional fundus photography captures only the central retina, limiting the assessment of peripheral lesions such as WWOP. Advancements in ultra-widefield (UWF) imaging have greatly advanced our understanding of the spatial distribution patterns of WWOP (Yu et al., 2022; Chan et al., 2025; Ludwig et al., 2021). By capturing up to 200 degrees of the retina in a single shot, UWF imaging overcomes the limitations of traditional fundus cameras and facilitates detection of anterior peripheral pathology in myopes (Ludwig et al., 2021). Studies consistently identify axial elongation as the strongest independent risk factor for WWOP, a mechanism closely linked to increased mechanical stress on the peripheral retina due to ocular expansion (Chen et al., 2018; Chan et al., 2025). This stress may manifest as vitreoretinal traction and peripheral hypoperfusion, contributing to WWOP formation and its frequent co-localization with other degenerations, such as lattice degeneration, in the temporal quadrants (Chan et al., 2025; Ludwig et al., 2021). Despite these improved observational capabilities, current clinical assessment of WWOP still heavily relies on subjective observation and empirical judgment, leaving its potential as an objective, quantifiable biomarker for myopic stress largely untapped. This limitation severely restricts the clinical utility of WWOP for risk stratification in myopia progression.

Artificial intelligence (AI), particularly deep learning (DL), offers a transformative solution. Deep convolutional neural networks (CNNs), a DL variant optimized for visual data analysis, have achieved diagnostic performance comparable to clinical experts in detecting and quantifying retinal diseases such as diabetic retinopathy and age-related macular degeneration (Ramesh et al., 2004; Poplin et al., 2018; Grzybowski et al., 2024; Dong et al., 2021). Furthermore, deep learning approaches have been extended to address specific challenges in ultra-widefield fundus imaging, such as the automated removal of eyelash artifacts that obscure peripheral retinal visualization (Xu and Yang, 2023). Building on this, deep learning has enabled precise, pixel-level segmentation of fundus structures, allowing for accurate delineation of lesion boundaries and quantitative feature extraction (Khatwani et al., 2022; Liu et al., 2022; Zunair and Ben Hamza, 2021; Wu et al., 2024; Wan et al., 2021). However, while deep learning-based segmentation has been successfully applied to macular pathologies such as diabetic retinopathy and age-related macular degeneration, WWOP presents unique challenges: ill-defined borders, peripheral location, and lack of standardized clinical grading. To our knowledge, no prior AI system has been developed specifically for automated quantification of WWOP.

Therefore, to address the existing clinical limitations, this study aimed to develop an AI-based system for the automatic segmentation and quantitative analysis of WWOP. Our work differs from existing approaches in three key aspects: (Syal et al., 2025): it targets a peripheral retinal lesion that is hypothesized to be associated with mechanical stress, rather than a macular pathology; (Karlin and Curtin, 1976); it introduces multi-dimensional quantification (area, count, zonal distribution) rather than binary detection; and (Fawzi et al., 2014) it provides a potential imaging biomarker for myopic mechanical stress, addressing a growing public health need in the context of the global myopia epidemic. This work aims to fill the current research gap in precise quantification and provide an innovative methodological tool for exploring its value as a key imaging biomarker in myopic retinopathy.

## 2 Materials and methods

### 2.1 Study design

This was a cross-sectional observational study with the primary objective of developing and validating an AI system for the quantitative analysis of WWOP. The study protocol was approved by the Institutional Ethics Committee (Approval No. 2026A-585), conducted in strict accordance with the principles of the Declaration of Helsinki, and written informed consent was obtained from all participants prior to enrollment.

### 2.2 Study participants

The study participants were recruited from individuals scheduled for refractive surgery (e.g., ICL implantation) or undergoing routine ophthalmic examinations. Inclusion criteria were individuals with myopia, defined as a spherical equivalent (SE) ≤ −0.50 D or an axial length (AL) ≥ 24.5 mm, with no age restriction. Exclusion criteria included significant media opacities affecting fundus image quality (e.g., severe cataract, corneal leukoma), a history of intraocular surgery (except uncomplicated cataract surgery performed more than 3 months prior with a stable postoperative state), and the presence of other retinal vascular diseases, macular disorders, or active uveitis that could interfere with WWOP assessment. Myopia severity was classified based on spherical equivalent (SE) as follows: low myopia (SE > −3.00 D and ≤ −0.50 D), moderate myopia (SE > −6.00 D and ≤ −3.00 D), and high myopia (SE ≤ −6.00 D), consistent with previous epidemiological studies (Yu et al., 2025; Wu et al., 2025; Flitcroft et al., 2019).

### 2.3 Image data acquisition

All participants underwent a comprehensive ophthalmic examination to collect baseline demographic and ocular characteristics. Ultra-widefield fundus photographs centered on the macula and optic disc were acquired using an Optos ultra-widefield camera (Optos plc, Dunfermline, United Kingdom), which captures up to 200 degrees of the retina in a single image. To ensure high and consistent image quality, photographs with significant artifacts, poor illumination, or media opacities affecting the visibility of the peripheral retina were excluded. Image acquisition followed a standardized protocol, and all images were reviewed by two experienced graders for quality control. The non-mydriatic widefield imaging system has been validated to show high agreement with traditional dilated fundus examination in detecting retinal pathology and was deemed suitable for the image acquisition needs of this study (Borrelli et al., 2020).

### 2.4 AI system development and quantitative analysis pipeline

The core pipeline of the developed AI system comprised three main steps: image preprocessing, lesion segmentation, and quantitative parameter extraction (Xu et al., 2019). First, standardized preprocessing was applied, including brightness and contrast adjustment, color space normalization, and background denoising, along with a rigid registration step based on the trajectory of the major vascular arcades to correct for image decentration caused by eye rotation. While the present system employed conventional preprocessing techniques, recent deep learning-based enhancement methods have shown promise in restoring degraded peripheral fundus images (Wan et al., 2021), epresenting a potential avenue for future pipeline refinement. For landmark identification, the AI system first automatically identified the optic disc center and the fovea as reference landmarks and computed the pixel distance between them, designated as the disc–fovea distance. For the lesion segmentation stage, a gold-standard dataset was established through pixel-wise manual annotation performed independently by two senior retinal specialists (He et al., 2024; Liu et al., 2023). For the lesion segmentation stage, a gold-standard dataset was established through pixel-wise manual annotation performed independently by two senior retinal specialists (with >10 years of experience in retinal imaging). The annotation criteria for WWOP were: (Syal et al., 2025): geographic, irregularly shaped white or gray-white areas in the peripheral retina; (Karlin and Curtin, 1976); absence of retinal depression or visible elevation; (Fawzi et al., 2014); no associated retinal breaks, holes, or detachment; and (Khatwani et al., 2022) clear demarcation from surrounding normal retina. These criteria were adapted from previously published definitions (Fawzi et al., 2014; Chan et al., 2025). Disagreements were resolved by consensus, with a third senior specialist adjudicating if necessary.

A total of 104 ultra-widefield fundus images from 104 myopic patients were used for model development. These images were randomly split into a training set (n = 74, 71.2%), a validation set (n = 15, 14.4%), and an internal test set (n = 15, 14.4%). The test set was completely independent and not used during any stage of model training or hyperparameter tuning. The reported performance metrics are based on this independent test set.

The U-Net-based segmentation model employed a ResNet-34 encoder pre-trained on ImageNet (Russakovsky et al., 2015). The loss function was a combination of binary cross-entropy and Dice loss (L = L_BCE + L_Dice). Training was performed using the Adam optimizer with an initial learning rate of 1 × 10^−4^, batch size of 8, and for a maximum of 200 epochs with early stopping (patience = 20) based on validation Dice coefficient. Data augmentation included random rotation (±180°), horizontal/vertical flips, elastic deformations (Simard et al., 2003), and contrast/brightness adjustments. All images were resized to 512 × 512 pixels. No external validation dataset from a different institution was available; therefore, the reported performance reflects internal test set evaluation only. Generalizability to other populations remains to be validated in future multi-center studies. A deep learning model based on the U-Net architecture was then trained on this dataset to achieve automatic segmentation (Zunair and Ben Hamza, 2021; He et al., 2024).

To ensure anatomical consistency of zonal division across images with varying axial lengths and gaze angles, a semi-automated, landmark-based partitioning strategy was employed. Specifically, the AI system first automatically identified the optic disc center and the fovea as reference landmarks and computed the pixel distance between them, designated as the disc–fovea distance. The posterior pole (R1) was defined as a circular region centered on the fovea with a radius of 1.5 times the disc–fovea distance. The mid-periphery (R2) was defined as an annular zone extending from 1.5 to 3.0 times the disc–fovea distance. The far periphery (R3) encompassed the remaining retinal area beyond 3.0 times the disc–fovea distance. This concentric zonal definition ensured consistent correspondence to the anatomical equator and ora serrata region.

Following automated segmentation of WWOP lesions, a comprehensive set of quantitative parameters was automatically calculated to characterize lesion burden and spatial distribution. All area measurements were derived from pixel counts using the formula:

$$\text{Area}\left(\;{\text{mm}}^{2}\right)=\left(\text{Area}\left(\text{pixels}\right)\times {\left(\text{Resolution}\right)}^{2}\right)\;/\;10^{6}$$

where Resolution is the pixel size in micrometers per pixel, as calibrated for the Optos ultra-widefield fundus camera. The core measurements included WWOP total area (mm^2^), defined as the total pixel area of all WWOP lesions converted to physical area; WWOP total number, representing the total number of discrete lesions detected as contiguous pixel clusters exceeding a minimum size threshold; and area ratio (%), calculated as (WWOP total area/total retinal area) × 100 to indicate the proportion of retina occupied by WWOP lesions.

**FIGURE 1:**
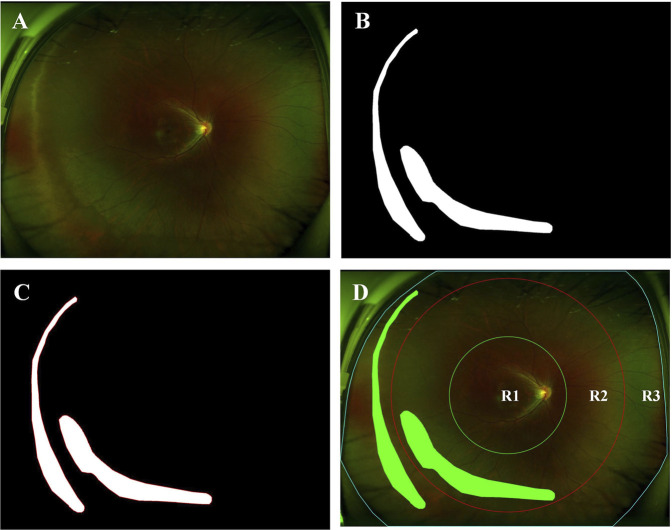
Workflow of WWOP annotation and zonal division for quantitative analysis. **(A)** Original ultra-widefield fundus photograph showing WWOP lesions. **(B)** Segmentation map of WWOP lesions generated by the AI model. **(C)** Delineation of WWOP lesion boundaries. **(D)** The same image with three concentric zones annotated (R1, R2, and R3, corresponding to the posterior pole, mid-periphery, and far periphery, respectively) and the WWOP lesion areas highlighted in green. These zones and lesion annotations were used to quantify the regional distribution and burden of WWOP.

The complete workflow is illustrated in Figure 1: the original ultra-widefield fundus photograph (Figure 1A) undergoes AI-based segmentation to generate a lesion map (Figure 1B) with clearly delineated boundaries (Figure 1C), followed by zonal annotation (Figure 1D) where WWOP lesions are highlighted in green and the three concentric zones (R1, R2, R3) are overlaid. For each zone, zonal area (Area R1/R2/R3) and zonal count (Number R1/R2/R3) were computed to quantify the total WWOP lesion area and the number of lesions within each specific zone. These zonal metrics enable precise localization of lesion burden and facilitate analysis of the relationship between axial elongation and the peripheral distribution of WWOP, transforming subjective visual assessment into objective, reproducible measurements suitable for longitudinal monitoring and risk stratification.

### 2.5 Statistical analysis

Statistical analysis was performed using SPSS software (version 27.0). Continuous variables are presented as mean ± standard deviation, while categorical variables are presented as frequencies and percentages. Inter-grader agreement for gold standard annotations was assessed using the Dice similarity coefficient and intraclass correlation coefficient (ICC) for total lesion area. For AI model performance evaluation, Dice similarity coefficient, Jaccard index, sensitivity, specificity, and precision were calculated by comparing automated segmentation results against the gold standard. Inter-group comparisons for baselinecharacteristics were conducted using one-way ANOVA (or Kruskal-Wallistest for non-normally distributed variables) for continuous variablesand the chi-square test for categorical variables, as appropriate. Spearman’s rank correlation coefficient was employed to assess thestrength of association between WWOP quantitative parameters andvariables such as axial length. Given the exploratory nature of thecorrelation analyses involving multiple WWOP parameters (nine parametersin Figure 3), we applied the Benjamini–Hochberg false discovery rate (FDR) procedure to control for type I error. All correlations reportedas statistically significant remained significant after FDR correction (q < 0.05). To assess the independent association between myopiaseverity and WWOP prevalence after adjusting for potential confounders, multivariable logistic regression was performed with WWOP presence asthe dependent variable, and age, sex, axial length, sphericalequivalent, K1, and K2 as independent variables. To further evaluate theindependent effect of axial length on WWOP burden, multiple linearregression analysis was performed with WWOP total area as the dependentvariable and axial length, age, and sex as independent variables. Variance inflation factor (VIF) was calculated to assessmulticollinearity. All statistical tests were two-tailed, with a P-value< 0.05 considered statistically significant.

## 3 Results

### 3.1 Baseline characteristics of participants

This cross-sectional study included 119 eyes from 119 participants with myopia, who were classified into three groups based on disease severity: low myopia (n = 13), moderate myopia (n = 44), and high myopia (n = 62). The baseline demographic and ocular characteristics of the participants are presented in Table 1. The overall cohort had a mean age of 23.96 ± 8.43 years, with intergroup variation: 28.62 ± 9.61 years for low myopia, 26.75 ± 8.29 years for moderate myopia, and 22.15 ± 7.93 years for high myopia; age differed significantly across groups (p < 0.001), with high myopia patients being younger. Regarding sex distribution, females constituted a higher proportion in the low and moderate myopia groups (61.54% and 61.36%, respectively), whereas the high myopia group showed a slightly higher proportion of males (45.16% vs. 54.84% females); however, sex distribution did not differ significantly across groups (p = 0.74). Mean BMI was 21.5 ± 1.3 kg/m^2^ in the low myopia group, 21.0 ± 1.8 kg/m^2^ in the moderate myopia group, and 22.1 ± 3.2 kg/m^2^ in the high myopia group, with no significant difference (p = 0.14). Ocular parameters reflected increasing myopic severity: mean axial length (AL) was 24.87 ± 1.17 mm in the low myopia group, 25.61 ± 1.05 mm in the moderate myopia group, and 27.24 ± 1.09 mm in the high myopia group (p < 0.001); corresponding mean spherical equivalent (SE) values were −1.64 ± 0.62 D, −4.38 ± 0.80 D, and −9.65 ± 2.82 D, respectively (p < 0.001). Mean keratometry readings (K1/K2) also demonstrated progressive steepening from low to high myopia: 41.57 ± 1.78/42.75 ± 1.82 D for low, 42.37 ± 1.71/43.60 ± 1.74 D for moderate, and 42.60 ± 1.55/44.47 ± 1.65 D for high myopia. K1 did not differ significantly across groups (p = 0.078), whereas K2 showed a significant progressive steepening (p = 0.0021).

**TABLE 1:** Baseline demographic and ocular characteristics.

Parameter	Low myopia (n = 13)	Moderate myopia (n = 44)	High myopia (n = 62)	P value
Male (%)	38.46	38.64	45.16	0.74
Female (%)	61.54	61.36	54.84	
BMI (kg/m^2^)	21.5 ± 1.3	21.0 ± 1.8	22.1 ± 3.2	0.14
Age (years)	28.62 ± 9.61	26.75 ± 8.29	22.15 ± 7.93	<0.001
AL (mm)	24.87 ± 1.17	25.61 ± 1.05	27.24 ± 1.09	<0.001
SE (D)	−1.64 ± 0.62	−4.38 ± 0.80	−9.65 ± 2.82	<0.001
K1 (D)	41.57 ± 1.78	42.37 ± 1.71	42.60 ± 1.55	0.078
K2 (D)	42.75 ± 1.82	43.60 ± 1.74	44.47 ± 1.65	0.0021

Abbreviations: SD, standard deviation; BMI, body mass index; AL, axial length; SE, spherical equivalent; K1/K2, corneal curvature.

### 3.2 Prevalence of WWOP lesions and representative fundus images

**FIGURE 2:**
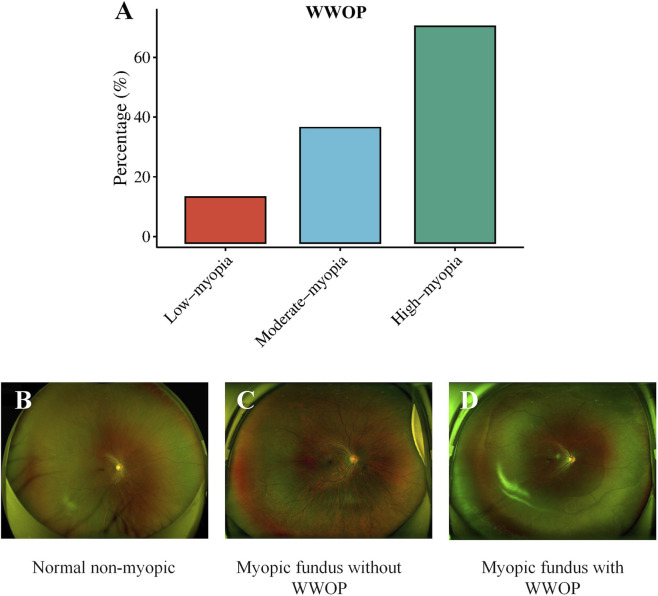
Prevalence of WWOP and representative fundus images. **(A)** Bar chart showing the proportion of eyes with and without WWOP lesions among the 119 myopic participants, stratified by myopia severity. **(B)** Ultra-widefield fundus photograph of a normal non-myopic eye. **(C)** Ultra-widefield fundus photograph of a myopic eye without WWOP. **(D)** Ultra-widefield fundus photograph of a myopic eye with WWOP lesions. These images illustrate the typical appearance of WWOP and its absence in myopic and non-myopic eyes.

The prevalence of WWOP lesions across different myopia severity groups is presented in Table 2. Among the 119 myopic eyes, the proportion of eyes with WWOP increased progressively with myopia severity: 15.38% (2/13) in the low myopia group, 38.64% (17/44) in the moderate myopia group, and 72.58% (45/62) in the high myopia group. Conversely, the proportion of eyes without WWOP was 84.62% (11/13), 61.36% (27/44), and 27.42% (17/62), respectively. This trend is visually depicted in the bar chart (Figure 2A), which shows the proportions of eyes with and without WWOP stratified by myopia severity. Representative ultra-widefield fundus images are shown in Figures 2B–D: (B) a normal non-myopic eye, (C) a myopic eye without WWOP, and (D) a myopic eye with WWOP lesions.

**TABLE 2:** Proportion of eyes with WWOP present and absent among low, moderate, and high myopia groups.

Group	WWOP present (%) (n/N)	WWOP absent (%) (n/N)
Low myopia	15.38% (2/13)	84.62% (11/13)
Moderate myopia	38.64% (17/44)	61.36% (27/44)
High myopia	72.58% (45/62)	27.42% (17/62)

To assess the independent association between myopia severity and WWOP prevalence after adjusting for potential confounders, multivariable logistic regression was performed with WWOP presence as the dependent variable, and age, sex, axial length, spherical equivalent, K1, and K2 as independent variables. The results are summarized in Table 3. After adjusting for all confounding factors (age, sex, axial length, spherical equivalent, K1, and K2), age remained a significant independent protective factor for WWOP occurrence: for each 1-year increase in age, the risk of WWOP decreased by 7.9% (OR = 0.921, 95% CI: 0.867–0.978, p = 0.007). None of the other variables reached statistical significance in this fully adjusted model.

**TABLE 3:** Multivariable logistic regression analysis for WWOP presence.

Variable	Regression coefficient	OR	95% CI lower	95% CI upper	P value
Age	−0.083	0.921	0.867	0.978	0.007
Sex	0.355	1.426	0.534	3.808	0.48
AL	0.427	1.532	0.633	3.706	0.34
SE	−0.158	0.854	0.600	1.215	0.38
K1	0.009	1.009	0.522	1.948	0.98
K2	−0.059	0.942	0.504	1.762	0.85

### 3.3 Performance evaluation of the AI system

The gold standard for WWOP segmentation was established by two senior retinal specialists. Prior to consensus, the inter-grader agreement was assessed on a random subset of 30 ultra-widefield fundus images. Both specialists independently annotated the WWOP lesions, and the agreement was evaluated using the Dice similarity coefficient and intraclass correlation coefficient (ICC) for lesion area measurements. The results demonstrated excellent agreement, with a mean Dice coefficient of 0.95 (95% CI: 0.93–0.97) and an ICC of 0.98 (95% CI: 0.96–0.99) for the total lesion area (Table 4). Discrepancies were resolved by consensus to establish the final pixel-wise gold standard.

**TABLE 4:** Inter-Grader Agreement for Gold Standard Annotations (n = 30 images).

Metric	Value (95% confidence interval)
Dice similarity coefficient	0.95 (0.93–0.97)
Intraclass correlation coefficient (ICC) for total lesion area	0.98 (0.96–0.99)
Mean absolute difference in total lesion area (mm^2^)	2.34 ± 1.12

On an independent test set comprising 15 ultra-widefield fundus photographs from 15 patients not involved in model training, the diagnostic performance of the AI model was systematically evaluated by comparing its automated segmentation results against this gold standard. The evaluation demonstrated that the model achieved a Dice similarity coefficient of 0.69, a Jaccard index of 0.53, sensitivity of 89.4%, specificity of 97.9%, and precision of 97.7%. The key performance metrics are summarized in Table 5.

**TABLE 5:** Performance metrics of the AI model on the independent test set.

Metric	Calculation formula	Model performance
Dice similarity coefficient	2 TP/(2 TP + FP + FN)	0.69
Sensitivity	TP/(TP + FN)	89.4%
Specificity	TN/(TN + FP)	97.9%
Precision	TP/(TP + FP)	97.7%
Jaccard index (IoU)	TP/(TP + FP + FN)	0.53

TP, true positive; FP, false positive; TN, true negative; FN, false negative.

### 3.4 Quantitative characteristics of WWOP and their correlations

Quantitative analysis using the AI system delineated the spatial and burden characteristics of WWOP lesions among the 64 participants with WWOP (2 low myopia, 17 moderate myopia, 45 high myopia). Notably, over 96% of the total lesion area was located outside the posterior pole, with the combined Area R2 (mid-peripheral area) and Area R3 (peripheral area) accounting for the vast majority (low myopia: 100%; moderate myopia: 96.8%; high myopia: 96.9% based on mean area ratio). Correspondingly, Number R3 (peripheral count) was consistently the highest among the zonal counts across all myopia groups (low myopia: 3.50 ± 2.12; moderate myopia: 2.29 ± 1.83; high myopia: 2.98 ± 2.39). Spatial distribution analysis based on lesion location and distribution pattern indicated a predominant involvement of the mid-periphery and periphery, as summarized in Table 6. Due to the small sample size of the low myopia group with WWOP (n = 2), these values are presented for descriptive purposes only and were excluded from statistical comparisons.

**TABLE 6:** Quantitative characteristics of WWOP by myopia severity.

Parameter category	Specific metric	Low myopia	Moderate myopia	High myopia
Zonal area	Area R1 (mm^2^)	0.00 ± 0.00	1.76 ± 6.11	1.63 ± 5.14
	Area R2 (mm^2^)	4.38 ± 6.20	17.81 ± 20.35	20.04 ± 26.93
	Area R3 (mm^2^)	41.19 ± 10.63	34.62 ± 27.39	30.43 ± 28.53
Zonal count	Number R1	0.00 ± 0.00	0.29 ± 0.69	0.31 ± 0.60
	Number R2	0.50 ± 0.71	1.53 ± 1.42	2.16 ± 1.88
	Number R3	3.50 ± 2.12	2.29 ± 1.83	2.98 ± 2.39
Global metrics	Total area (mm^2^)	45.57 ± 4.44	54.24 ± 37.84	52.14 ± 40.43
	Total number	3.00 ± 1.41	2.35 ± 1.58	3.02 ± 1.94
Relative metric	Area ratio (%)	4.75 ± 0.78	4.32 ± 2.98	4.86 ± 3.50

**FIGURE 3:**
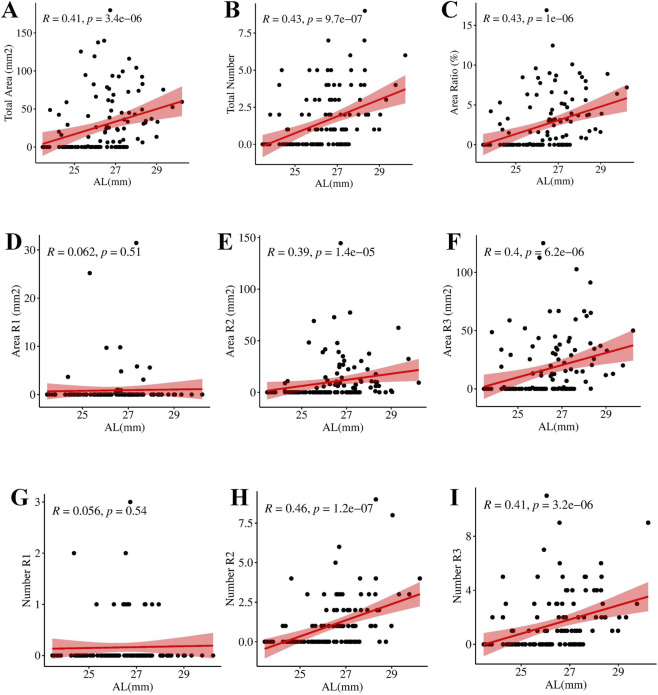
Scatter plots of WWOP parameters versus axial length. Each panel **(A–I)** shows the correlation between axial length (AL, mm) and a specific WWOP metric: **(A)** Total Area, **(B)** Total Number, **(C)** Area Ratio, **(D)** Area R1, **(E)** Area R2, **(F)** Area R3, **(G)** Number R1, **(H)** Number R2, and **(I)** Number R3. Spearman’s correlation coefficient (R) and P-value are displayed in each plot. The solid line represents the linear regression fit for visual guidance.

Correlation analysis revealed significant positive associations between several WWOP burden parameters and axial length. Spearman’s rank correlation coefficients showed that WWOP Total Area (R = 0.41, P < 0.001) and Total Number (R = 0.43, P < 0.001) increased significantly with longer axial length. At the zonal level, Area R3 (peripheral area) exhibited a significant positive correlation (R = 0.40, P < 0.001), while Area R2 (mid-peripheral area) also showed a significant correlation (R = 0.39, P < 0.001). Notably, Number R2 (mid-peripheral count) demonstrated the strongest correlation of all parameters (R = 0.46, P < 0.001), suggesting that this region may be particularly sensitive to axial elongation. A comprehensive visualization of all quantitative parameters and their correlations is presented in Figure 3. All reported correlations remained statistically significant after Benjamini–Hochberg FDR correction (q < 0.05; see Table 7).

**TABLE 7:** Spearman correlation analysis between WWOP metrics and axial length (with FDR correction).

Metric	Spearman’s R	Raw P-value	Corrected q-value	Significant? (q < 0.05)
Total area (mm^2^)	0.41	<0.0001	0.0000	Yes
Total number	0.43	<0.0001	0.0000	Yes
Area ratio (%)	0.43	<0.0001	0.0000	Yes
Area R1 (mm^2^)	0.062	0.51	0.54	No
Area R2 (mm^2^)	0.39	<0.0001	0.0000	Yes
Area R3 (mm^2^)	0.40	<0.0001	0.0000	Yes
Number R1	0.056	0.54	0.54	No
Number R2	0.46	<0.0001	0.0000	Yes
Number R3	0.41	<0.0001	0.0000	Yes

Multiple linear regression analysis was conducted on the full cohort of 119 eyes to assess the independent contributions of axial length, age, and sex to WWOP total area. The overall model was statistically significant (*R* ^2^ = 0.112, adjusted *R* ^2^ = 0.089; F (3, 115) = 4.838, P = 0.003). After adjusting for age and sex, axial length remained a significant independent predictor of WWOP total area (standardized coefficient β = 0.274; unstandardized B = 7.46 mm^2^ per mm increase in AL; 95% confidence interval: 2.47–12.45; P = 0.004). Neither age (β = −0.152, P = 0.117) nor sex (β = −0.042, P = 0.664) demonstrated significant independent associations. All variance inflation factors were below 1.2, indicating negligible multicollinearity. Although statistically significant, the model explained only 11.2% of the variance in WWOP total area, suggesting that other unmeasured factors (e.g., vitreoretinal traction, peripheral perfusion status) may contribute substantially to WWOP burden. These findings confirm that the positive association between axial elongation and WWOP burden is independent of demographic confounders, further supporting the hypothesis that mechanical stretching constitutes the primary pathogenic driver of WWOP formation.

## 4 Discussion

This study successfully developed and preliminarily validated a fully automated, AI-based quantitative analysis system for White Without Pressure (WWOP). By enabling objective, high-precision measurement of lesion burden, zonal distribution, and spatial patterns, our system overcomes the longstanding limitations of subjective clinical assessment and provides a transformative tool for WWOP research and clinical practice.

First, the system revealed a preferential distribution of WWOP in the peripheral retina and its predominant involvement of the mid-periphery and far periphery (R2/R3), which reconfirms observations based on ultra-widefield imaging by Chan et al. (Chan et al., 2025) and Khatwani et al. (Khatwani et al., 2022) and provides more robust support through precise quantitative data. Our finding aligns with recent large-scale studies confirming that WWOP and dark without pressure (DWOP) lesions are predominantly located in the temporal retina (Chan et al., 2025). This pattern aligns with regional anatomical vulnerabilities and the effects of vitreoretinal traction during ocular elongation (Chan et al., 2025; Ludwig et al., 2021).

Second, our study provides compelling quantitative evidence linking axial elongation to WWOP burden. Significant positive correlations between axial length and multiple WWOP parameters, including total area (R = 0.41, P < 0.001), peripheral area R3 (R = 0.4, P < 0.001), and total number (R = 0.43, P < 0.001), directly support the pathological hypothesis that mechanical stretch from axial elongation is the core mechanism for WWOP formation (Chen et al., 2018; Chan et al., 2025). Notably, the mid-peripheral lesion count (Number R2) exhibited the strongest correlation (R = 0.46, P < 0.001), suggesting that this region may serve as a sensitive early indicator of mechanical stress. This biomechanical link is further supported by multimodal imaging studies showing peripheral vascular abnormalities and nonperfusion areas that worsen with longer axial lengths (Ludwig et al., 2021), as well as outer retinal thinning and reduced deep vascular complex perfusion in eyes with co-occurring Dark Without Pressure (Yu et al., 2022). Together, these findings establish WWOP as a quantifiable imaging correlate of axial elongation-related mechanical stress, positioning it as a candidate imaging biomarker for myopic progression.

Third, the quantitative metrics derived from our system provide a quantifiable link between axial elongation and peripheral retinal degeneration risk. Our finding of an association between a higher WWOP area ratio and a greater likelihood of peripheral retinal degeneration aligns with the established understanding that axial elongation elevates the risk of lattice degeneration and retinal breaks (Chen et al., 2018). This is consistent with population-level data showing drastically increased odds of peripheral retinal changes in high myopia (Zhang et al., 2018), collectively suggesting that quantitative WWOP assessment could serve as an objective, quantifiable imaging biomarker for identifying “high-risk” myopic eyes.

Fourth, regarding the potential confounding effect of age, our multivariable logistic regression analysis (adjusting for age, sex, axial length, spherical equivalent, K1, and K2) revealed that age was a significant independent protective factor for WWOP occurrence (OR = 0.921 per 1-year increase, 95% CI: 0.867–0.978, p = 0.007). This indicates that younger age is associated with higher WWOP prevalence, a finding consistent with recent large-scale ultra-widefield imaging studies that have also demonstrated a significant association between younger age and a greater number of affected sectors of white and dark without pressure lesions in myopic eyes (Chan et al., 2025; Brennan et al., 2024). Although the high myopia group was significantly younger than the low myopia group (p < 0.001), which could theoretically bias the prevalence estimate if age were a risk factor, the fully adjusted model confirmed that the effect of age is independent and opposite to the direction of myopia severity. In other words, the higher WWOP prevalence in high myopia cannot be explained by age differences; instead, age itself appears to have a protective effect. This finding may reflect the cumulative mechanical stress during active axial elongation in younger individuals, as axial elongation rates are highest in younger age groups and decrease progressively with increasing age (Jonas et al., 2023; Chen et al., 2025; McMonnies, 2016), and it underscores the importance of adjusting for age when comparing WWOP burden across myopia groups. Future longitudinal studies are needed to determine whether WWOP lesions regress or stabilize with increasing age.

The demonstrated capabilities of this AI system translate into tangible clinical and research utility. In clinical practice, its high reproducibility and objectivity overcome the variability and ambiguity inherent in traditional subjective assessment, enabling precise comparison of images acquired at different time points or by different examiners, thus providing a reliable tool for monitoring the dynamic evolution of WWOP during longitudinal follow-up. In scientific research, the finely extracted zonal parameters (e.g., Area R3 and Number R3) offer a new data dimension for exploring the relationship between WWOP and biomechanical stress in specific retinal regions. Future research can build upon this, incorporating baseline axial elongation rates and other information to construct predictive models for the earlier identification of high-risk myopic individuals (Du et al., 2024). Additionally, future iterations could aim to integrate with other imaging modalities, such as widefield OCT/OCTA, to correlate WWOP burden with microstructural changes (e.g., outer retinal thickness) and perfusion metrics, providing a more comprehensive risk assessment (Yu et al., 2022; Ludwig et al., 2021).

Regarding our model’s segmentation performance (Dice = 0.69), we acknowledge that this is moderate compared to values typically reported for well-demarcated macular lesions (e.g., 0.85–0.95 for optic disc or DR lesions) (Kwon et al., 2018). However, it is comparable to prior studies segmenting peripheral or ill-defined retinal lesions. For example, similar Dice values (0.65–0.75) have been reported for choroidal neovascularization segmentation on OCT and for peripheral drusen (Zantut et al., 2021). More importantly, the high specificity (97.9%) and precision (97.7%) indicate that false positives are rare, which is clinically desirable for screening applications where over-calling WWOP could lead to unnecessary follow-up (Sun et al., 2023). The moderate Dice value reflects the inherent difficulty of WWOP segmentation, i.e., its subtle, irregular, and low-contrast appearance, rather than model inadequacy. Nevertheless, we acknowledge that further improvements (e.g., incorporating attention mechanisms or multi-modal imaging) are warranted to enhance segmentation accuracy before clinical deployment.

While this study presents a novel tool and insights, several limitations must be acknowledged to contextualize the findings and guide future work. Firstly, the cross-sectional design limits causal inference. Future long-term, prospective cohort studies are needed for validation. Secondly, the study population was relatively young (mean age 23.96 years) and recruited from a single refractive surgery center in China. This may limit generalizability to older populations, non-Asianethnicities, or community-based myopic patients. The prevalence andquantitative characteristics of WWOP may differ with age, ethnicity,and environmental factors. Therefore, external validation inmulti-center, diverse populations is essential before clinicalimplementation. Thirdly, the current system relies entirely ontwo-dimensional analysis of color fundus photographs, lacking assessmentof retinal microstructure and blood flow perfusion. Furthermore, the AImodel was developed and tested on images from a single camera type (Optos) without external validation; its generalizability to otherultra-widefield devices remains unknown. Subsequent work could exploreintegration with technologies like optical coherence tomography. Fourthly, the sample size, while sufficient for the primary analyses,may have limited the power to detect weaker associations (e.g., theeffect of sex) and precluded subgroup analyses by myopia severity ordetailed quadrant-level modeling. Finally, the AI model’s Dicecoefficient of 0.69, while acceptable for research purposes, requiresfurther improvement before clinical deployment.

## 5 Conclusion

In conclusion, we have developed an AI system that enables accurate, automated segmentation and multi-dimensional quantitative analysis of White Without Pressure. Applying it to 119 myopic eyes (64 with WWOP) confirmed the characteristic peripheral (mid-periphery and far periphery) distribution of WWOP and revealed significant positive correlations between axial length and both total lesion area (R = 0.41, P < 0.001) and total lesion number (R = 0.43, P < 0.001). These findings support axial elongation as a key determinant of WWOP burden and underscore its potential as an objective, quantifiable imaging biomarker for myopic stress. However, given the cross-sectional design, the moderate segmentation Dice coefficient (0.69), and the lack of external validation, the system should currently be considered a research tool rather than a ready-for-clinical-deployment solution. Future prospective, multi-center, and longitudinal studies are required to establish its role in risk stratification and personalized management of high myopia.

## Data Availability

The original contributions presented in the study are included in the article/supplementary material, further inquiries can be directed to the corresponding author.
